# Lasers and Energy Devices for the Skin: Conventional and Unconventional Use

**DOI:** 10.1155/2016/9031091

**Published:** 2016-11-09

**Authors:** Steven Paul Nistico, Andrea Chiricozzi, Federica Tamburi, Giovanni Cannarozzo

**Affiliations:** ^1^Department of Health Sciences, University of Catanzaro, Catanzaro, Italy; ^2^Unit of Dermatology, University of Rome Tor Vergata, Rome, Italy; ^3^Unit of Dermatology, University of Pisa, Pisa, Italy; ^4^Unit of Dermatology, Catholic University of Rome, Rome, Italy

Laser technology represents nowadays an important advance in dermatology. Physical, technological, and clinical research is currently carried out in order to optimize laser-skin interaction and laser efficacy-safety profile.

The different dermatological indications may be summarized following the different spectral features of the laser sources used:

- UV laser and light sources: they have been used primarily for the treatment of inflammatory skin diseases and/or vitiligo, as well as striae. The mechanism of action is immunomodulatory. The XeCl excimer laser emits at 308 nm, near the peak action spectrum for psoriasis. Other UV nonlaser sources like the 355 nm have also been used for vitiligo and hypopigmentation disorders and various inflammatory diseases. The paper by N. Zerbinati et al. contributes to explaining the mechanism of action of the 355 nm laser.
- Violet IPL spectra and low-power 410 nm LED and fluorescent lamps: both are used either alone or with ALA. Alone, the devices take advantage of endogenous porphyrins and kill* P. acnes*. After application of ALA, this wavelength range is highly effective in creating singlet O_2_ after absorption by PpIX. Uses include treatment of AKs, actinic cheilitis, and BCCs.
- Near-IR(A) (595, 755, and 810 nm): these wavelengths are used primarily to treat blood vessels and hyperpigmented lesions. They are positioned in the absorption spectrum for blood and melanin and will penetrate deeply enough to treat vessels up to 2 mm. Newer lasers, such as the Pulsed Dye 595 nm, may be indicated for the treatment of Port Wine Stains and infantile haemangiomas. Q switched lasers in this spectrum may be useful to treat multicoloured tattoos. The article by our group covers the nonconventional dermatological applications of this wavelength.
- Near-IR(B) 940 and 1064 nm: these two wavelengths have been used extensively for lager and deeper blood vessels on the legs and face. Because of the depth of penetration (on the order of millimeters), they are especially useful in coagulation of deeper blood vessels and selective follicle denaturation for safe and effective hair removal. Q switched 1064 nm lasers are very effective on dark ink tattoos.
- Medium-IR lasers (1320–1540 nm): they heat tissue water, shrink collagen, and are widely used in cosmetology for antiageing purposes, treatment of striae, and acne scarring (nonablative fractional procedures).
- Far-IR systems: represented mainly by the CO_2_ and Er:YAG lasers. Dermatological applications are mainly surgical (warts, dermal nevi) and cosmetological thanks to their precision in ablation. Fractional CO_2_ lasers guarantee a very precise epidermal and dermal heating that makes this device ideal for facial skin resurfacing (fine or moderate wrinkles, dyspigmentation, and acne scarring) on facial skin. A novel application on keratosis pilaris has been herein covered by V. Vachiramon. Newer devices combine lasers to other energy sources such as radiofrequency in order to optimize antiageing dermatological procedures. In this special issue, these nonlaser systems are deeply covered (W. Manuskiatti et al. and D. H. Kim et al.).

By compiling these papers, we hope to enrich our readers and researchers with respect to the laser technology in the field of dermatology and cosmetology.

*Steven Paul Nistico* *Andrea Chiricozzi* *Federica Tamburi* *Giovanni Cannarozzo*

